# Endogenous Retroviruses Transcriptional Modulation After Severe Infection, Trauma and Burn

**DOI:** 10.3389/fimmu.2018.03091

**Published:** 2019-01-08

**Authors:** Olivier Tabone, Marine Mommert, Camille Jourdan, Elisabeth Cerrato, Matthieu Legrand, Alain Lepape, Bernard Allaouchiche, Thomas Rimmelé, Alexandre Pachot, Guillaume Monneret, Fabienne Venet, François Mallet, Julien Textoris

**Affiliations:** ^1^EA7426 Hospices Civils de Lyon–bioMérieux–UCBL1 “Pathophysiology of Injury Induced Immunosuppression,” Groupement Hospitalier Edouard Herriot, Lyon, France; ^2^Joint Research Unit, Hospice Civils de Lyon, bioMerieux, Centre Hospitalier Lyon Sud, Pierre-Benite, France; ^3^Department of Anesthesiology and Critical Care and Burn Unit, Groupe Hospitalier St-Louis-Lariboisière, Assistance publique-Hôpitaux de Paris, Paris, France; ^4^Hospices Civils de Lyon, Intensive Care Unit, Centre Hospitalier Lyon Sud, Pierre Bénite, France; ^5^Agressions Pulmonaires et Circulatoires dans le Sepsis APCSe VetAgro Sup UPSP 2016.A101, Centre Hospitalier Lyon-Sud, Pierre Bénite, France; ^6^Hospices Civils de Lyon, Immunology Laboratory, Groupement Hospitalier Edouard Herriot, Lyon, France; ^7^Hospices Civils de Lyon, Department of Anaesthesiology and Critical Care Medicine, Groupement Hospitalier Edouard Herriot, Université Claude Bernard Lyon 1, Lyon, France

**Keywords:** endogenous retroviruses, severe inflammatory injuries, septic shock, burn, trauma, transcriptome, host response

## Abstract

Although human endogenous retroviruses (HERVs) expression is a growing subject of interest, no study focused before on specific endogenous retroviruses loci activation in severely injured patients. Yet, HERV reactivation is observed in immunity compromised settings like some cancers and auto-immune diseases. Our objective was to assess the transcriptional modulation of HERVs in burn, trauma and septic shock patients. We analyzed HERV transcriptome with microarray data from whole blood samples of a burn cohort (*n* = 30), a trauma cohort (*n* = 105) and 2 septic shock cohorts (*n* = 28, *n* = 51), and healthy volunteers (HV, *n* = 60). We described expression of the 337 probesets targeting HERV from U133 plus 2.0 microarray in each dataset and then we compared HERVs transcriptional modulation of patients compared to healthy volunteers. Although all 4 cohorts contained critically ill patients, the majority of the 337 HERVs was not expressed (around 74% in mean). Each cohort had differentially expressed probesets in patients compared to HV (from 19 to 46). Strikingly, 5 HERVs were in common in all types of severely injured patients, with 4 being up-modulated in patients. We highlighted co-expressed profiles between HERV and nearby CD55 and CD300LF genes as well as autonomous HERV expression. We suggest an inflammatory-specific HERV transcriptional response, and importantly, we introduce that the HERVs close to immunity-related genes might have a role on its expression.

## Introduction

Human Endogenous Retroviruses (HERVs) are former exogenous retroviruses which have infected germinal cells and became integrated in our genome million years ago (1). These rare events happened several times in evolution. As retrotransposons, they are able to duplicate across the genome and they represent today more than 8% of our genome. Each insertion therefore led to distinct groups or families, each including multiple copies. Current classification annotates around 100 such groups.

HERV loci initially shared a common structure with exogenous retroviruses: internal protein coding regions (*gag, pro, pol, env*) flanked by two identical Long Terminal Repeats (LTRs). The accumulation of mutations and recombination events during evolution made most of these elements incomplete and defective for replication. Indeed, most of HERVs in our genome are now solo LTRs (1) resulting from recombination between 5′ and 3′ proviral LTRs. LTRs are critical elements that control viral gene expression either as promoters, enhancers or as polyadenylation signals. When inserted upstream, within or downstream of a “conventional” protein coding gene, LTRs can modulate its expression pattern (2, 3). For example, the presence of intronic LTR can result in novel transcripts, by providing alternative promoters, enhancers or polyadenylation signals, or by altering RNA splicing (4–6). Very few is known about of the transcriptional modulation of such elements in pathological contexts but in cancers [like testicular cancer (7) or colorectal cancer (8)] and auto-immune diseases [like multiple sclerosis (9–11)].

Few studies focused on HERVs reactivation in acute inflammatory contexts. In mice, modulation of HERVs expression has been shown to be quite specific, with signatures related to pathogen-associated molecular pattern (PAMPs) (12). In human, LPS or PMA stimulations of myeloid cells revealed an increase expression of four HERVs families (13). *In vivo*, HERVs expression has been detected in the plasma and whole blood samples of burn patients (14, 15) although the studies focused on whole HERVs families, not on specific loci. Studying HERV transcriptome modulation after severe inflammatory injuries could help to better understand pathological states of patients.

After severe injuries like septic shock, burn or trauma, leading to an important inflammatory response, we and others have shown that the blood transcriptome is highly modulated, with early and profound changes in adaptive and innate immune responses (16, 17). Moreover, in these contexts, viral reactivation is often observed, especially for Herpes Viruses (18, 19). This reactivation is associated with an immunosuppressive state (20). We therefore hypothesize that HERV, like latent viruses, may reactivate and be transcribed *in vivo* after inflammatory injuries. Given that several groups showed that some probes of commercial whole genome microarray do target HERV loci (21, 22) (such as Affymetrix U133 plus 2), we retrospectively explored microarray datasets obtained in our lab to study the HERV transcriptome modulation in various contexts of injuries *in vivo*.

## Materials and Methods

### Patients and Sample Collection

#### Microarray Analyzed Cohort

##### Burns cohort

30 severe burn patients admitted at Hospices Civils de Lyon, France (HCL) were included in a placebo-controlled, randomized, double-blind study assessing the efficacy of hydrocortisone administration on burn shock duration. Inclusion / exclusion criteria, clinical description and ethical considerations of the cohort have been previously published elsewhere (16, 23). Thirteen healthy volunteers were also recruited within Hospices Civils de Lyon to serve as controls for the transcriptional study. Whole blood samples were collected at inclusion (severe shock, before any treatment, Day 1) and in the following days (around day 2 (D2), day 5 (D5), and day 7 (D7) after inclusion).

##### Traumas cohort

105 patients with severe trauma were admitted at HCL. Briefly, patients were included when they were under mechanic ventilation, with an Injury Severity Score (ISS) over 25 and were at least 18 years old. Inclusion / exclusion criteria and ethical considerations of the cohort have been previously published elsewhere (24). The main clinical variables are summarized on Table S1. Samples were collected at day 1 (D1) or day 2 (D2) after trauma. Data from 22 healthy volunteers were also used to make comparisons with patients (identical with septic shock cohort 2).

##### Septic shock cohort 1(SS1)

Twenty-eight septic shock patients and 25 HV admitted into 2 ICUs of HCL were included in this study to explore the early transcriptome modulation after septic shock. Inclusion/exclusion criteria, clinical description and ethical considerations of the cohort have been previously published elsewhere (25). The first blood sample was collected at the onset of shock (i.e., within 30 min after the beginning of vasoactive treatment, D0) and at day 1 (D1) and day 2 (D2) after shock.

##### Septic shock cohort 2 (SS2)

Fifty-one septic shock patients admitted to two Intensive Care Units (ICU) of HCL and 22 HV were included in a prognostic biomarker study. Inclusion / exclusion criteria, clinical description and ethical considerations of the cohort have been previously published elsewhere (26). Samples were collected at day 1 (D1), day 2 (D2) and day 3 (D3) after shock.

#### RT-qPCR Validation Cohorts

##### Patients

Subset of cohorts used for microarray analysis were used for validation cohort: 10 burn samples at D1, 10 traumas samples at D1, 10 SS1 samples at D1, 10 SS2 samples at D1. Each subset was matched with its corresponding cohort on: Age, sex and Total Burn Surface Area (TBSA) for burns-Sex, Sepsis at D7 and Death at D28 for traumas-Age, sex and SAPS II for SS1-Age, Sex and Death at D28 for SS2.

##### Healthy Volunteers

Whole blood samples were purchased from the Etablissement Français du Sang (*n* = 12). The mean age of HV is 56, with a standard error of 9. According to the standardized procedure for blood donation, written informed consent was obtained from healthy volunteers (HVs) and personal data for blood donors were anonymized at time of blood donation and before blood transfer to a research lab.

#### Flow Cytometry Validation Cohort

##### Burns

Whole blood samples (EDTA tubes) from 13 burn patients sampled at D1 and D7 and admitted in Edouard Herriot hospital at Lyon, France were recruited as part of the EARLYBURN study (NCT02940171). Patients were aged from 21 to 84 (mean = 53), 12 men. The mean TBSA was 33% (from 20 to 52%). All samples from these patients were used for CD300LF protein analysis, and 7 of these 13 patients were used for *CD55* protein analysis.

##### Septic shocks

Whole blood samples (EDTA tubes) from 22 septic shock patients sampled at D1/D2, D3/D4/D5, and D6/D7/D8 after shock and admitted in Edouard Herriot hospital at Lyon, France were recruited as part of IMMUNOSEPSIS study (NCT02803346). Patients were aged from 23 to 81 (mean = 68), 16 men. Eleven samples were used for CD300LF protein analysis and 11 other samples for *CD55* protein analysis.

##### Healthy volunteers

Whole blood samples (EDTA tubes) were purchased from the Etablissement Français du Sang (*n* = 18). Donors were aged from 21 to 63 (mean = 50), 12 men and 6 women. They were age-matched with burn and septic shock cohorts. According to the standardized procedure for blood donation, written informed consent was obtained from healthy volunteers (HVs) and personal data for blood donors were anonymized at time of blood donation and before blood transfer to a research lab.

### RNA Extraction and Microarrays

Total RNA was extracted with PAXgene™ Blood RNA kit (PreAnalytix, Hilden, Germany). Whole blood from PAXGene™ tubes was preferred to either buffy coat or PBMCs to ensure reproducibility and avoid missing samples within the context of a clinical study. RNA integrity was assessed using Agilent 2100 Bioanalyser (Agilent Technologies, Waldbrom, Germany) and Lab-on-chip RNA 6000 Nano Assay (Agilent Technologies). Double-stranded cDNA was prepared from total RNA and an oligo-dT primer using GeneChip One-Cycle cDNA Synthesis Kit (Affymetrix, Santa Clara, United States). Three microgram labeled cRNA were hybridized onto Human Genome U133 Plus 2.0 GeneChips (Affymetrix), revealed and washed using FS450 fluidic station. GeneChips were scanned using a 5G scanner (Affymetrix) and images (DAT files) were converted to CEL files using GCOS software (Affymetrix).

### Microarray Analysis

Microarray data are available on the Gene Expression Omnibus (GEO) website for Burn [GEO:GSE77791], SS1 [GEO:GSE57065], and SS2 [GEO:GSE95233] cohorts. The preprocessing methods were comparable in all datasets. Microarray normalization and statistical analysis were performed using R/Bioconductor (R v3.2.3). Quality assessment was performed through simpleaffy (v2.46.0) (27). After removing outlier samples the raw data were normalized, adjusted for background noise and summarized using the GCRMA (Guanine Cytosine Robust Multi-Array) algorithm with default parameters (28). COMBAT algorithm (29) was used to remove batch effect on Burn and Trauma cohorts. The 337 probesets from the U133 Plus2.0 microarray targeting HERVs have been identified and selected as described elsewhere (21, 22).

All the analysis were made with R (3.2.3). The differential expression analysis was performed with Limma package (3.26.9) (30). A probeset was considered significantly statistically differentially expressed between two conditions when absolute log2 Fold Change was higher than 0.5 and adjusted *P*-values [Benjamini-Hochberg correction (31)] lower than 0.01.

### Reverse Transcription and Quantitative PCR

RNA from the cohorts, according to the above criteria, and new RNA from HV were selected. RNA concentration was determined using Quant-iT RNA, BR assay on Qubit (Life Technologies, Chicago, Ilinois, United States). RNA integrity was assessed with the RNA 6000 Nano Kit on a Bioanalyzer (Agilent Technologies, Santa Clara, California, United States). Samples with RNA integrity number ≤6 were excluded due to poor quality RNA. Total RNA was reverse transcribed in complementary DNA (200 ng in a final volume of 20 μL) using QuantiTect Reverse Transcription kit (Qiagen) as recommended by the manufacturer. The expression levels of genes (*CD55, CD300LF, SLC8A1, NFE4, PTTG1IP*, and *HPRT1* as reference gene) and associated HERVs were quantified using quantitative-real time polymerase chain reaction (qPCR). qPCR were performed on a LightCycler instrument using Light Cycler 480 Probes Master for the genes and reference genes and on SYBR Green I master for HERVs. Final volume of 20 μL contains 0.5 μM of primers. For genes, an initial denaturation step of 10 min at 95°C followed by 45 cycles, 10 s at 95°C, 29 s annealing at 60°C, and 1 s extension at 72°C, Taqman) was performed. For HERVs, an initial denaturation step of 5 min at 95°C followed by 45 cycles of a PCR protocol (10 s at 95°C, 15 s at 55°C and 15 s at 72°C, SYBR Green program), melting curve protocol was performed. The Second Derivative Maximum Method was used with the LightCycler software (Release 1.5.1) to automatically determine the crossing point for individual samples. Standard curves were generated by using serial dilutions of cDNA standards prepared from purified PCR amplicons obtained with the corresponding primers (Table S2). Relative standard curves describing the PCR efficiency of selected targets were created and used to perform efficiency-corrected quantification with the LightCycler Relative Quantification Software. Targets expression normalization was performed using a selected housekeeping gene (hypoxanthine phosphoribosyltransferase 1 [HPRT1, (32)]), and results were expressed as normalized concentration ratio.

### Flow Cytometry

#### Sampling and Staining

The following antibodies were used: anti CD14-BV510, anti CD3–BV421 and anti CD56–PECy7 from BD Biosciences; anti CD300lf-PE from BD Biosciences or anti *CD55*-APC from Biolegend; anti CD16-APC from BD Biosciences or anti CD16-FITC from Beckman Coulter (Miami, FL) and PE Mouse IgG1, κ Isotype Control from BD Biosciences or APC Mouse IgG1, κ Isotype Control from R&D System. Red blood cell lysis was performed using Versalyse lysing solution (Beckman Coulter). *CD300LF* and *CD55* expression were measured using Navios flow cytometer (Beckman-Coulter). Results were analyzed with Kaluza software (Beckman-Coulter) expressed as Medians of Fluorescence Intensity (MFI).

### Statistics

Wilcoxon signed rank tests were done for RT-qPCR and flow cytometry results, by comparison between HV and each cohort of patients, for each target.

## Ethics Approval and Consent to Participate

EDTA blood tubes were obtained from EFS (Etablissement Français du Sang) and used immediately. In accordance with EFS standardized procedures for blood donation, written no-objection was obtained from healthy volunteers to use the blood for the research and personal data for blood donors were anonymized before blood transfer to our research lab.

Protocols of the discovery and validation cohorts were approved by local ethics committees. Non-opposition to inclusion in the protocols was systematically recorded from patients or next of kin.

## Results

We studied the *in vivo* modulation of the HERV transcriptome in three clinical relevant models of acute inflammatory injury: a burn, a trauma and 2 septic shock cohorts. We analyzed expression from each cohort independently comparing patients with healthy volunteers. All cohorts included severely injured patients (Table 1). The 30 burn patients had a median total burn surface area (TBSA) of 70% and high severity scores [median Baux: 110, median Abbreviated Burn Severity Index (ABSI): 11]. The 105 trauma patients had a median Injury Severity Score (ISS) score of 34 and a median Simplified Acute Physiology Score II (SAPSII) of 44. The 28 septic shocks from SS1 cohort had a median SAPSII of 45 and a median Charlson score of 2. The 51 patients from SS2 cohort had a median SAPSII of 51.

**Table 1:** Patients characteristics of burn, trauma, and septic shock cohorts included in microarray analyses.

**Variable**	**Burn**	**Trauma**	**Septic shock 1**	**Septic shock 2**
	**(*n =* 30)**	**(*n =* 105)**	**(*n =* 28)**	**(*n =* 51)**
Age, years	48 (39–55)	38 (25–54)	62 (54–76)	65 (53–74)
Gender, women, n (%)	8 (27%)	34 (32%)	9 (32%)	18 (35%)
Weight, kg	94 (77–104)	78 (67–92)	–	–
TBSA (%)	70 (48–84)	–	–	–
Baux score	110 (102–125)	–	–	–
ABSI score	11 (10–12)	–	–	–
ISS score	–	34 (29–41)	–	–
SOFA score	–	5 (1–7)	10 (9–13)	10 (8–12)
SAPSII score	–	44 (29–56)	45 (34–56)	51 (43–62)
Secondary septic shock	12 (40%)	29 (28%)	–	–
ICU length of stay, days	66 (22–89)	9 (5–17)	10 (5–14)	–
D28 mortality, n (%)	8 (27%)	4 (4%)	5 (18%)	17 (33%)

*TBSA, Total Burn Surface Area (severe patient > 30%); Baux score, Predictor of mortality due to severe burns (severe patient > 100); ABSI, Abbreviated Burn Severity Index (severe patient > 9); ISS, Injury Severity Score (severe patient > 15); SOFA, Sequential organ failure assessment score (severe patient ≥ 3); SAPSII, Simplified Acute Physiology Score II. (severe patient > 30). In each cohort, cumulative mortality has been assessed up to the 28^th^ day after ICU admission*.

**Figure 1:**
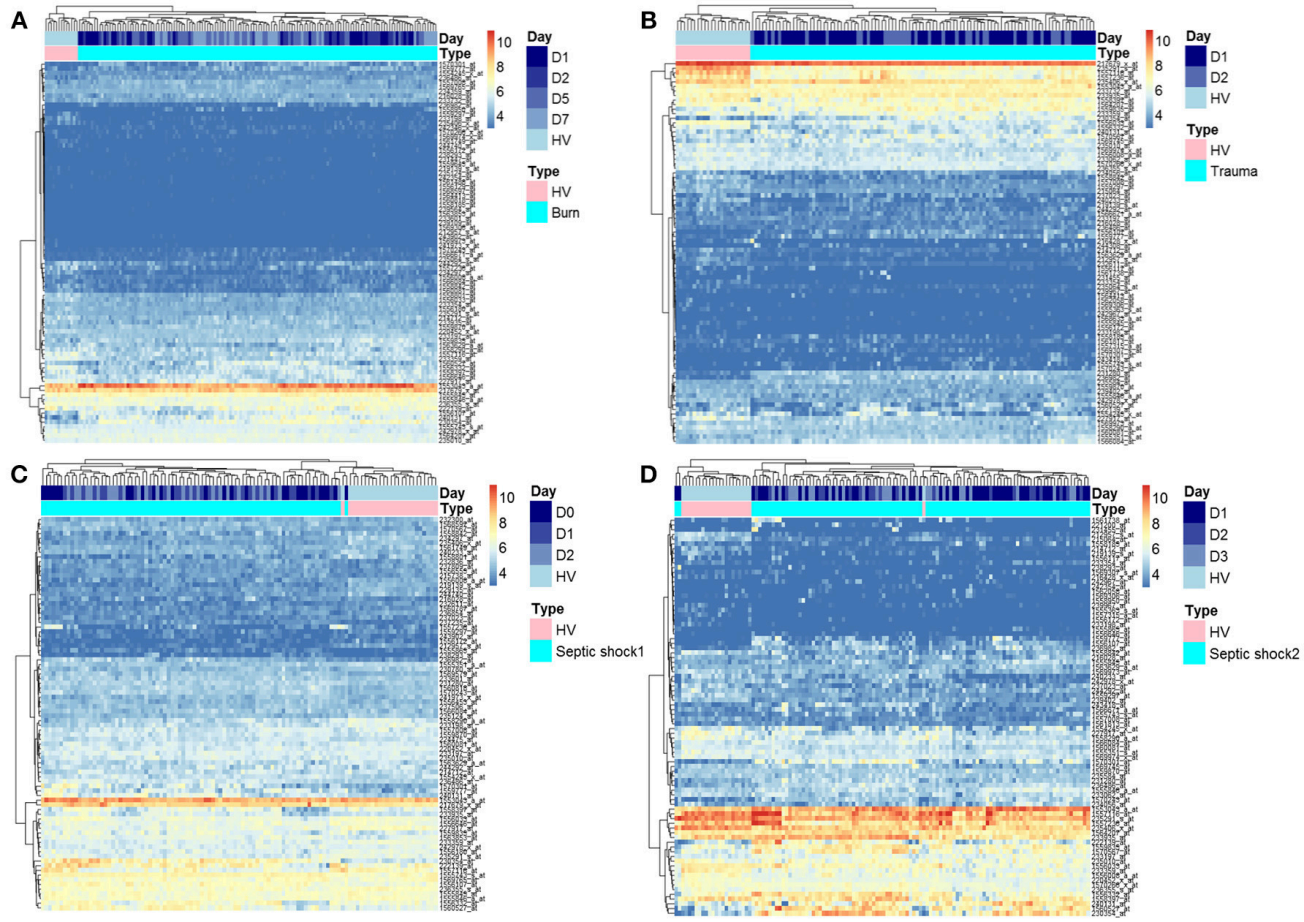
Heatmap representation of HERVs in three models of injury. Heatmap of the 25% most variant probesets targeting HERVs in the four datasets: burn, trauma, and 2 septic shock cohorts. Probesets are in rows and samples in columns. Samples are annotated (colored bars on the top) by type of samples (HV in pink, patients in cyan) and day after inclusion (blue scaled). Expression levels are color-coded from blue (low expression) to red (high expression). Similar patterns of expression are highlighted through hierarchical clustering of probesets (rows) and samples (columns) with Euclidean distance and complete clustering method. **(A)** Expression levels in burn patients. **(B)** Expression levels in trauma patients. **(C)** Expression levels in septic shock 1 patients. **(D)** Expression levels in septic shock 2 patients.

**Figure 2:**
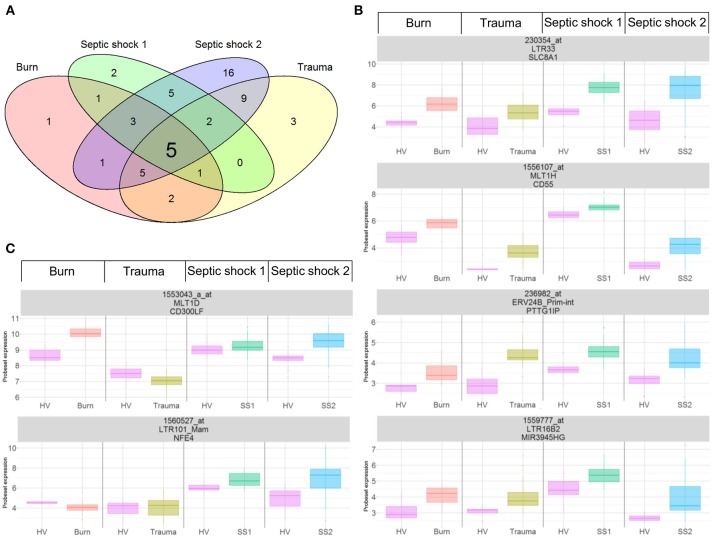
Differentially expressed HERVs in severely injured patients. **(A)** Venn diagram of differentially expressed HERVs for each dataset. **(B)** Expression profiles of commonly modulated probesets targeting HERVs in the 4 datasets, at D1. **(C)** Expression profiles of 2 selected probesets targeting HERVs. Boxes are color-coded by cohort. For each graphic, from top to bottom, title contains: probeset name, HERV name and closest gene.

As previously published (21, 22), we extracted data from 337 probesets targeting HERVs loci from the whole genome U133 plus 2.0 microarray datasets. Among them, a majority had low expression levels, within background levels (Supplemental Figure 1). Based on hierarchical clustering analysis, 64 probesets (19%) were expressed (i.e., above background) for burns, 60 probesets (18%) for traumas, 164 for septic shock 1 (49%) and 63 for septic shocks 2 (19%). The 25% most variant probesets (*n* = 84) across samples in each dataset revealed that several probesets were even highly expressed (Figure 1). In each dataset, the hierarchical clustering highlighted a clear difference between patients and HV, suggesting a modulation of HERV expression following injury. Interestingly, over these top 25% most variant probesets selected in each dataset (resulting of 127 distinct probesets), 44 (35%) were similarly modulated in the four datasets, and 102 (80%) in at least 2 datasets (Supplemental Figure 2). In order to analyze the HERV transcriptome modulation associated with injury, we performed a supervised analysis comparing HERV expression in injured patients at D1 (admission) and HV, in each dataset separately. The comparison (accounting for multiple testing correction with absolute fold change higher than 1 and corrected *p*-value lower than 0.01) between burn patients and HV resulted in 19 differentially expressed HERVs (Supplemental Figure 3A). The comparison between trauma patients and HV resulted in 27 differentially expressed HERVs (Supplemental Figure 3B). The comparison between septic shock patients and HV resulted in 19 and 46 differentially expressed HERVs for cohorts 1 and 2 respectively (Supplemental Figures 3C,D). Altogether, 56 distinct probesets targeting HERVs were differentially expressed among all 4 datasets, clearly discriminating HV from patients at ICU admission (Supplemental Figure 4 and Table S3). Taking into account the global profile for each probeset, 16 (28.6%) had higher expression in patients compared to HV and 40 (71.4%) were down-modulated in patients. Interestingly, 5 probesets were differentially expressed in all 4 datasets and 16 in at least 3 of them (Figure 2A). All 5 commonly modulated probesets had consistent expression profile across the 4 datasets. Four were over-expressed in patients compared to healthy volunteers (Figure 2B). The 5th probeset, down-modulated in all datasets, maps at multiple locations in the genome and was not considered in further analyses. Among the 4 remaining modulated probesets, 1 HERV from ERV24B_Prim-int family (236982_at), is within 2kb from the *PTTG1IP* gene and 3 are within a gene. A HERV from LTR33 family (230354_at) is within an intron of *SLC8A1* gene. A HERV from MLT1H family (1556107_at) and one from LTR16B2 family (1559777_at) are located in the 3'UTR of *CD55* and *MIR3945HG* genes respectively (Table 2).

**Table 2:** Genomic and transcriptomic features of the 6 probesets of interest.

	**HERV Probesets^a^**					
**Transcriptomic & genomic features**	**1556107_at**	**230354_at**	**1553043_a_at**	**1560527_at**	**1559777_at**	**236982_at**
Patients vs. HVs	UP	UP	UP (for Burn & SS2)	UP (for SS1 & SS2)	UP	UP
log2FC^b^ in						
*Burn*	1.13	1.73	1.48	−0.55	1.05	0.77
*Trauma*	1.31	1.47	−0.33	1.50	0.79	1.57
*SS1*	0.57	2.03	0.26	0.72	1.07	0.90
*SS2*	1.45	2.97	1.08	2.00	1.26	1.12
Confirmed^c^	Yes^*^	Yes^*^	Yes^*^	Yes^*^	No	No
HERV family	MLT1H	LTR33	MLT1D	LTR101_Mam	LTR16B2	ERV24B_Prim-int
HERV coordinates^d^	chr1 207372720-207272854	chr2 40545338-40545778	chr17 74694268-74694744	chr7 102988743-102988923	chr4 184844993-184845324	chr21 44875454-44876122
Closest gene	CD55	SLC8A1	CD300LF	NFE4	MIR3945HG	PTTG1IP
Localization^e^	3′ UTR	intron 1	3′UTR	3′end	3′ UTR	promoter region

a *HGU133plus2 Affymetrix probesets mapping on a HERV locus*.

b *A positive log2 FC means that the probeset is more expressed in patients than in HV*.

c *Confirmed by RT-qPCR*.

* *Expression confirmed. Modulation between patients and HV not always statistically confirmed, mainly due to high inter-individual variability*.

d *Grch38 genomic coordinates of HERV locus*.

e *Localization of HERV element according to the nearby gene*.

Moreover, we selected 2 other probesets of interest(1553043_a_at and 1560527_at, Figure 2C). The first one targets a MLT1D HERV located in the 3′UTR of *CD300LF*. It was up-modulated in burn and SS2 cohorts. It had a strong up-modulation at D1 in burn patients compared to HV, decreasing over the first week toward HV expression level at D7 (Supplemental Figure 5). The second one targets a LTR101_Mam HERV located in a 3′UTR of a processed transcripts of *NFE4* gene. It was differentially expressed in the 2 septic shock cohorts. This probeset had the highest log2FC among the 5 septic shock-specific modulated probesets.

**Figure 3:**
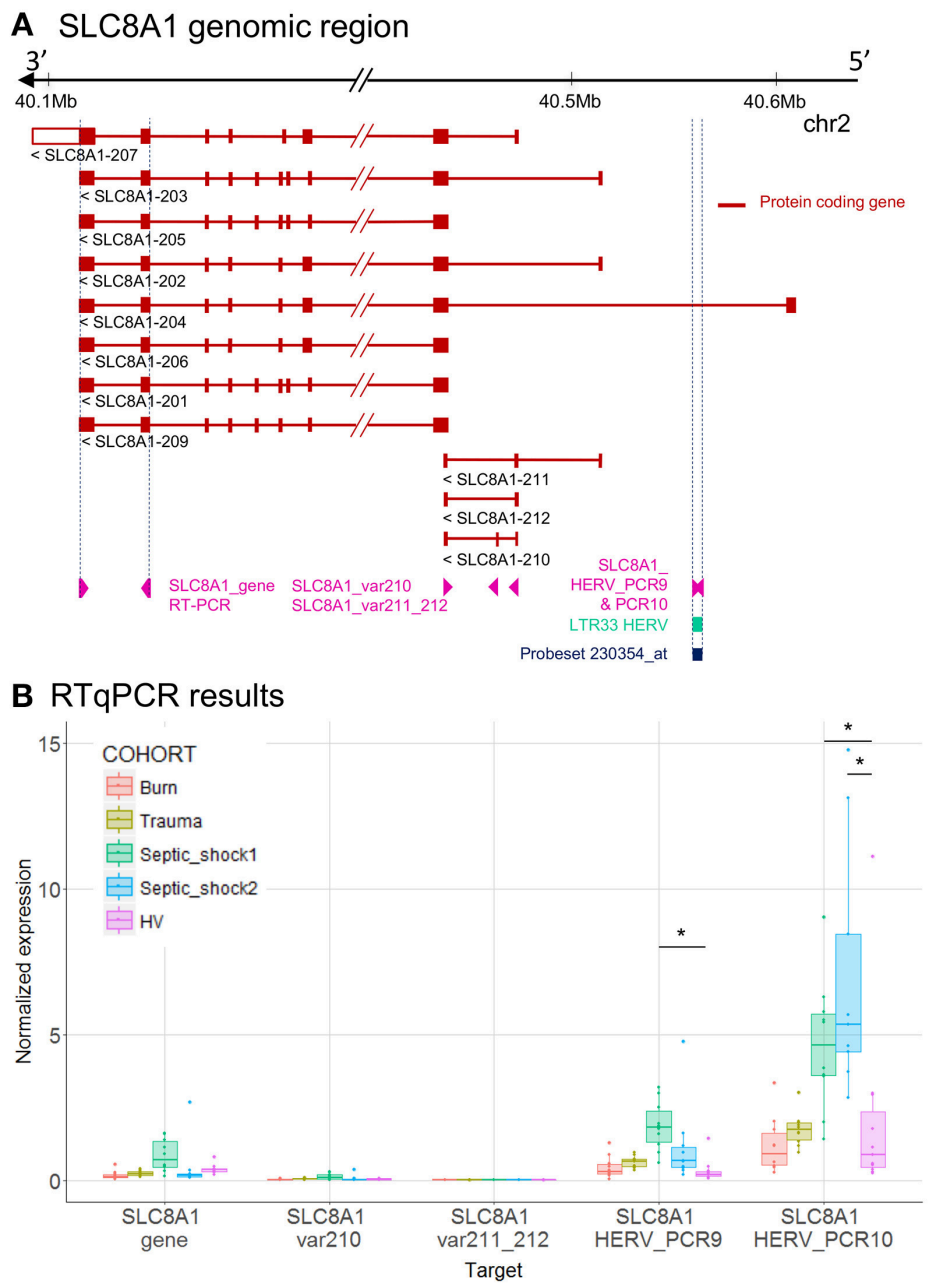
LTR33 HERV and *SLC8A1* gene expression. **(A)** *SLC8A1* genomic region, with the position of HERV in green, probeset in dark blue, and PCR designs in purple. **(B)** Expression levels of specific transcripts by RT-qPCR, as described in A, in HV and patients at D1. Expression levels (copy number / μl) were normalized with reference gene (*HPRT1*). Boxes are color-coded by cohort. Statistically significant difference with HV is marked by * (Wilcoxon signed rank test, *p* < 0.05).

**Figure 4:**
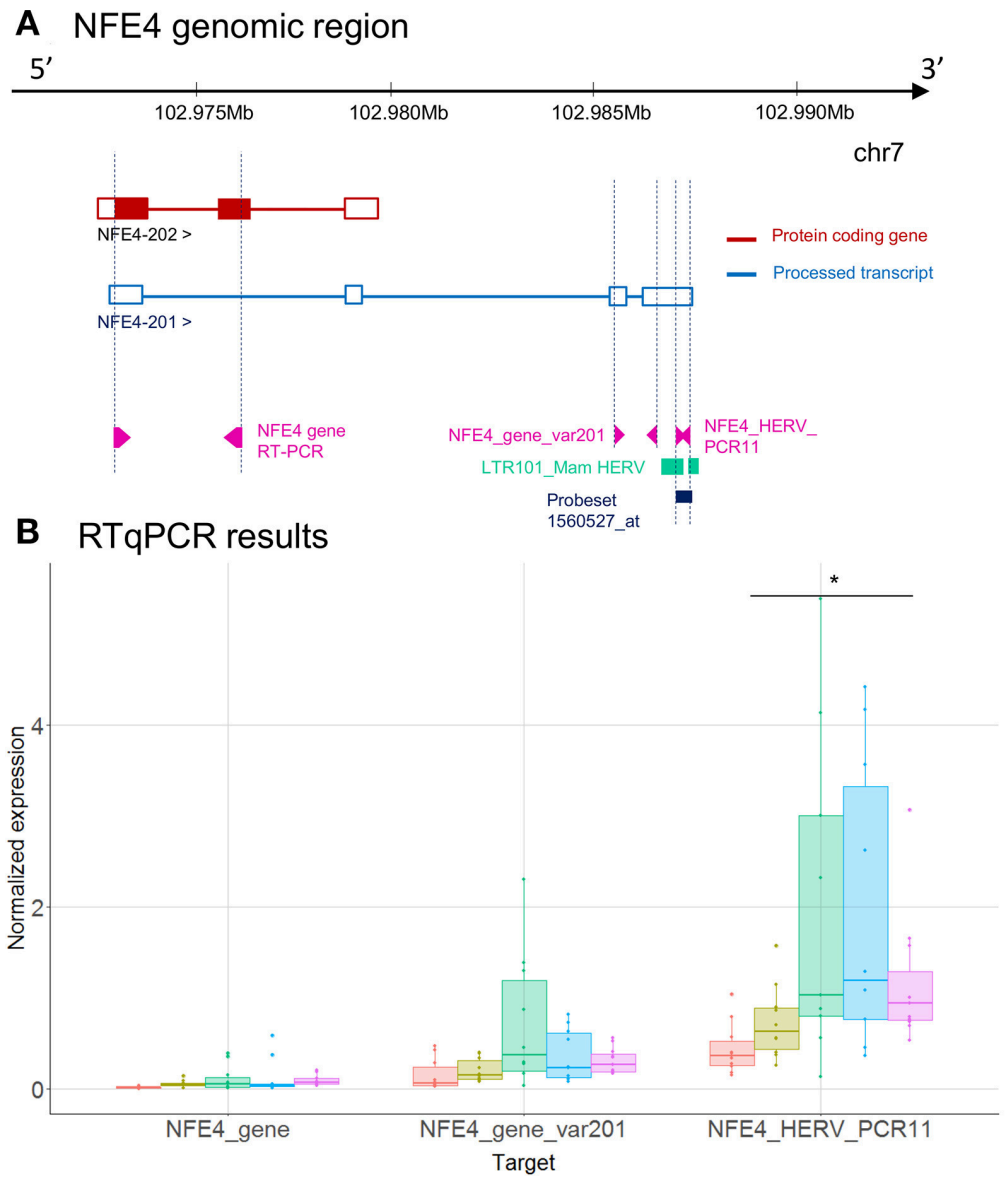
LTR101_Mam HERV and *NFE4* gene expression. **(A)** *NFE4* genomic region, with the position of HERV in green, of probeset in dark blue, of PCR designs in purple. **(B)** Expression levels of specific transcripts by RT-qPCR, as described in A, in HV and patients at D1. Expression levels (copy number / μl) were normalized with reference gene (*HPRT1*). Boxes are color-coded by cohort. Statistically significant difference with HV is marked by * (Wilcoxon signed rank test, *p* < 0.05).

**Figure 5:**
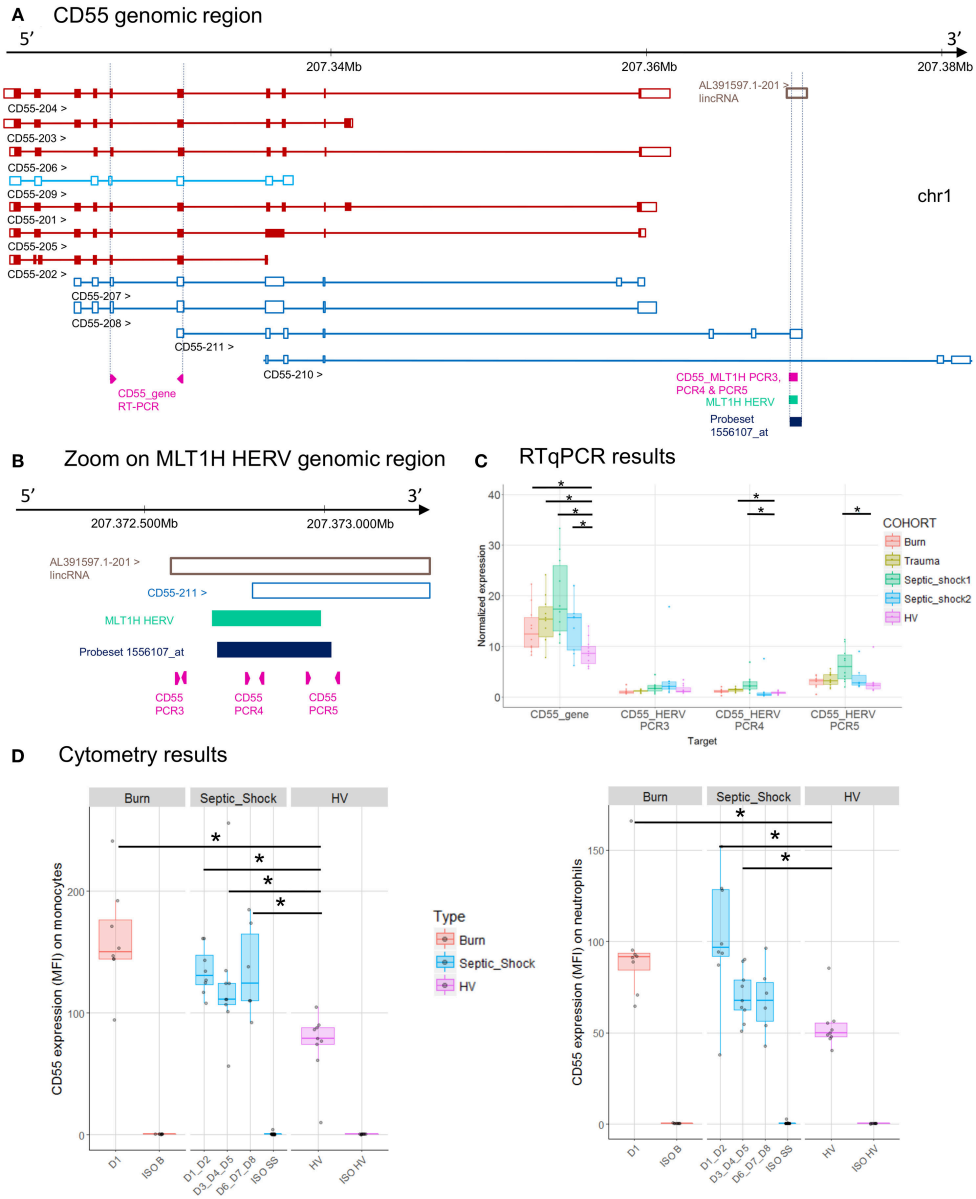
*CD55* associated HERV. **(A)** *CD55* genomic region, with the positions of HERV in green, of probeset in dark blue, of PCR designs in purple. **(B)** Zoom in genomic region of HERV showing PCR designs in detail. **(C)** Expression levels of specific transcripts by RT-qPCR, as described in A and B, in HV and patients at D1. Expression levels (copy number/μl) were normalized with reference gene (*HPRT1*). Boxes are color-coded by cohort. **(D)** Protein expression levels (MFI), on monocytes (left) and neutrophils (right) from 8 burn patients (red), 11 septic shock patients (blue), and 9 HV (purple). Columns ISO B, ISO SS, and ISO HV correspond to isotypes for burn, septic shock, and HV, respectively. Statistically significant difference with HV is marked by * (Wilcoxon signed rank test, *p* < 0.05).

**Figure 6:**
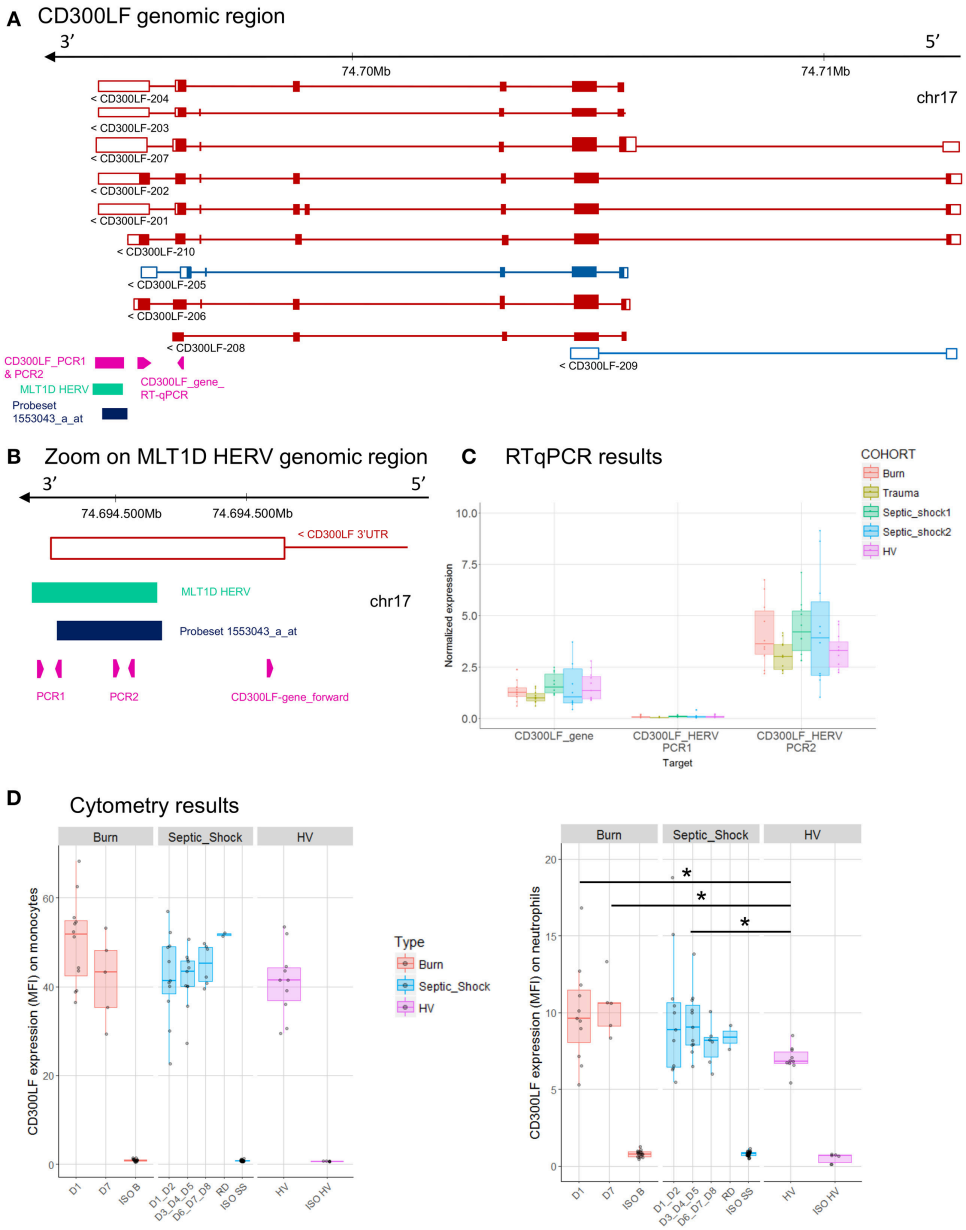
*CD300LF* associated HERV. **(A)** *CD300LF* genomic region, with the positions of HERV in green, of probeset in dark blue, of PCR designs in purple. **(B)** Zoom in genomic region of HERV showing PCR designs in detail. **(C)** Expression levels of specific transcripts by RT-qPCR, as described in A and B, in HV and patients at D1. Expression levels (copy number/μl) were normalized with reference gene (*HPRT1*). Boxes are color-coded by cohort. **(D)** Protein expression levels (MFI), on monocytes (left), and neutrophils (right) from 14 burn patients (red), 11 septic shock patients (blue), and 10 HV (purple). Columns ISO B, ISO SS, and ISO HV correspond to isotypes for burn, septic shock, and HV, respectively. Statistically significant difference with HV is marked by * (Wilcoxon signed rank test, *p* < 0.05).

To validate these transcriptional HERV modulations, we designed primers on the 6 described HERV loci above, and on nearby genes by RT-qPCR (Table S2). For each targeted region, we made multiple RT-qPCR designs. We identified several distinct patterns of expression comparing HERVs and nearby genes (Tables 2): (i) for *PTTG1IP* and *MIR3945HG* regions, we observed no or low signal from the HERV loci (data not shown), (ii) for SLC8A1 (Figure 3) and *NFE4* (Figure 4) regions, we observed a high signal from HERVs elements, but no or lower signal on the genes, (iii) for *CD55* (Figure 5) and *CD300LF* (Figure 6) regions, we observed a middle or high signal from both HERV loci and genes.

To better interpret the results, we extracted from Ensembl the genome annotation and showed in genomic context, the microarray and the RT-qPCR results of *SLC8A1* (Figure 3), *NFE4* (Figure 4), *CD55* (Figure 5) and *CD300LF* (Figure 6) regions. *SLC8A1* has 11 known transcripts. All but one are located in 3′ of the LTR33 HERV element targeted by the 230354_at probeset, which is located in the first intron of SLC8A1-204 transcript (Figure 3A). The up-modulation of the LTR33 element in septic shock patients observed on microarray was confirmed by RT-qPCR (Figures 2B, 3B). The up-modulation observed for other cohorts was not confirmed by RT-qPCR. The gene *SLC8A1* was not expressed in patients or HV, as seen on various microarray probesets and confirmed by RT-qPCR (SLC8A1_gene, var210, var211_212).

*NFE4* gene has 2 transcripts (Figure 4A) and only one is coding for a protein (NFE4-202). The LTR101_Mam HERV element, targeted by the 2560527_at probeset, is located in 3′UTR of NFE4-201, the non-protein-coding transcript. Although the same trends are observed between microarray and RT-qPCR, the up-modulation of the LTR101_Mam element observed in septic shock patients with microarray was not statistically significant in RT-qPCR (Figures 2C, 4B). There was low or no signal on designs targeting gene transcripts (NFE4_gene and NFE4_gene_var201).

*CD55* gene has 11 transcripts. The MLT1H HERV element, targeted by the 1556107_at probeset, is located in the 3′UTR of CD55-211 transcript (Figure 5A). The HERV element overlaps the 3′UTR of transcript CD55-211 and a long intergenic noncoding RNA (lincRNA, a class of long transcribed RNA molecules longer than 200 nucleotides and not coding for proteins) (Figure 5B). The up-modulation of MLT1H seen with microarray in the 4 cohorts was partially confirmed by RT-qPCR on trauma and septic shock cohorts (Figures 2B, 5C). The designs targeting MLT1H or close neighborhood (PCR3, 4 and 5) presented the same profile, with a significant difference in septic shock and trauma cohorts compared to HV (PCR4). The design targeting the gene showed also up-modulation of *CD55* and a very high absolute normalized expression in patients compared to HV (Figure 5C). [Of note 1555950_a_at probeset, targeting most of *CD55* transcripts, was also up-modulated in patients, and with a high expression level (data not shown)]. We also confirmed by flow cytometry on monocytes and neutrophils that *CD55* expression was higher in patients than in HV, confirming an up-modulation at the protein level in patients (Figure 5D).

The MLT1D HERV element, targeted by the 1553043_a_at probeset is located in 3′UTR of CD300LF-201, 202, 203, 204, and 207 protein-coding transcripts (Figure 6A). We made several RT-qPCR designs, targeting either the HERV locus only (PCR1) or both HERV and 3'UTR of *CD300LF* (PCR2, Figure 6B). We confirmed the expression of HERV locus, but the up-modulation seen in burn and septic shock 2 cohorts compared to HV on microarray was not confirmed by RT-qPCR, neither for gene nor for HERV designs (Figures 2C, 6C). PCR1 showed no signal at all. PCR2 design showed a slight higher expression level in burn and septic shock cohorts compared to HV. We also confirmed an higher expression at the protein level by flow cytometry on neutrophils in burn and septic shock patients, compared to HV (Figure 6D). In monocytes, protein level in burn patients at D1 seemed slightly higher than HV.

## Discussion

We took advantage of previous microarray analyses on four cohorts of severely injured patients to assess the modulation of HERV transcriptome in acute inflammation. We showed that several loci were expressed and modulated after acute injury. Surprisingly, a large majority among the modulated HERVs were down-modulated in patients compared to HV, whereas a global and massive gene up-modulation has been observed after severe injuries (17).

Five HERVs were modulated in patients compared to HV in all four datasets and 16 HERVs in at least 3 datasets, suggesting a similar inflammatory triggered modulation in all models. We validated expression profiles by RT-qPCR on 6 regions, allowing us to explore more precisely the modulation pattern of the HERVs and the neighbor genes. Interestingly, all these 6 HERVs have detected signals in RNAseq experiments from lymphoid cells and whole blood datasets [Ensembl Rnaseq tracks (33)]. Some authors already focused on HERV detection in blood of burn patients using pan-family RT-PCRs (14, 15).

Moreover, very few data are available in human diseases for specific loci. No study had yet evaluated the expression of HERVs in acute inflammatory contexts by using multiple cohorts with different types of inflammatory injuries.

Several groups showed that huge epigenetic modifications occur after acute inflammation, regulating transcriptional profiles in the immune system, especially in sepsis (34, 35). These epigenetic modifications may explain the polarization profiles such as tolerance or trained immunity, observed after various stimulations of innate cells (35). We hypothesized and confirmed *in vivo* that other elements than genes, especially HERVs which are known to be tightly controlled by epigenetic modifications (36), might be modulated in acute inflammatory situations. This has also been demonstrated in other pathophysiological contexts such as cancer (7, 8, 37, 38), where global epigenetic modifications are also observed (39, 40).

Interestingly in cancer, epigenetic modifications that gave access to HERV cis sequences through open chromatin, have also revealed a very role in pathophysiology (2, 5, 37). Indeed, by providing alternative promoter sequences to classical protein coding genes, these epigenetic modifications explain part of the ectopic expression of myeloid-growth factor receptors in lymphoid cells (37). Recently, it has been suggested that HERV could provide transcription factor binding sites, modulating immune-related gene expression, as illustrated by MER41 elements which bring STAT1 motifs to IFNγ inducible genes (41). An exhaustive study on HERV expression with a different tool, like CHIPseq technology, would bring valuable data to find potential TFBS on HERV sequences. This underlines how HERV elements, in particular their LTRs, could modulate gene expression and the host immune response to injury. In our study, the four commonly modulated HERVs were LTRs located nearby genes related to the immune response. In several cases (*NFE4, CD300LF*), we found a polyadenylation signal (AAUAAA) provided by the HERV LTR in 3′ of some of the alternative transcripts of the genes. The case of *CD300LF* is interesting as this protein acts as an inhibitory receptor for myeloid cells (42). The LTR might stabilize specific transcripts and enhance expression of CD300LF protein, which we confirmed by flow cytometry in severe burn patients early after admission. This up regulation might participate in the compensatory anti-inflammatory response. The precise understanding of the mechanisms through which specific HERV LTRs might impact immune gene expression is not possible in such translational research setting with patient samples. This will require in the future *in vitro* experimental models to validate and understand our observations.

Our RT-qPCR validation assays also showed inter-individual variability and underlined that exploring such repertoire of our genome, repetitive sequences, may face specificity issues, and will require specific tools. Indeed, as a first attempt, we used commercial microarray where probesets were not initially designed to target HERV elements. Moreover, as the probesets targeting HERVs were initially supposed to target conventional genes, the majority of explored HERVs are close to or within a gene. To better understand HERV expression in these settings, targeting HERVs localized far from genes seems important. Until now, the lack of tool made difficult the exploration of HERV expression. It would be interesting to reproduce these analyses, with a more exhaustive technology designed to specifically target HERVs, like the HERV-V3 Affymetrix microarray we recently published (43), or even RNAseq. It will allow us to better describe the whole HERV transcriptome modulation and understand the putative global role of HERV in the host response.

Finally, it would be of importance to consider HERV expression in further blood transcriptome analyses, especially in such acute inflammatory contexts, to better understand HERV expression during host response. Such studies, based on well-defined cohorts including criteria for patient stratification and taking into account drug treatment, should allow to estimate whether HERVs could be good markers of the different immune phases that follow acute injury. More, whether HERV could become potential therapeutic targets would basically require to decipher which circulating cell type produces which HERV. It will thus be appropriate to develop dedicated cellular models to, in one hand better understand the contribution of each blood-cell type to HERV expression and in another hand how HERV expression may contribute to the cell response to stimuli. To conclude, we showed for the first time that specific HERV loci are transcribed in whole blood of ICU patients. Our design allowed us to identify specific transcriptional signatures of HERVs elements, *in vivo*, linked to the acute inflammatory response. Moreover, the similarities observed in three models of acute injuries suggest common regulatory mechanisms and a specificity of the observed modulation. We also unravel the potential regulatory role of these elements within the host immune response. Further studies are needed to better understand such mechanisms and how HERVs may contribute to the pathophysiology of the host immune response, a key part of the pathophysiology of sepsis.

## Data Availability Statement

Microarray expression data has been deposited on NCBI Gene Expression Omnibus and are accessible through GEO accession numbers GEO:GSE77791, GEO:GSE57065 and GEO:GSE95233. Data from microarray experiment for trauma cohort are available at Hospices Civils de Lyon—bioMérieux—UCBL1 “Pathophysiology of Injury Induced Immunosuppression,” Groupement Hospitalier Edouard Herriot, France.

## Author Contributions

OT, FM and JT designed the project, performed the analyses and wrote the paper. CJ and FV performed cytometry experiments. EC performed RT-qPCR validations. ML, AL, BA, and TR recruited patients in the various cohorts. OT, MM, FV, FM, and JT read and discussed the manuscript. All authors drafted or revised critically the manuscript for important intellectual contents. All authors read and approved the final manuscript.

### Conflict of Interest Statement

OT, MM, CJ, EC, AP, FM, and JT are employees of an *in-vitro* diagnostic company. The remaining authors declare that the research was conducted in the absence of any commercial or financial relationships that could be construed as a potential conflict of interest.

## Supplementary Material

Supplemental Figure 1 Heatmap representation of HERVs in three models of injury. Heatmap of the 337 probesets targeting HERVs in the four datasets: burn, trauma and 2 septic shock cohorts. Probesets are in rows and samples in columns. Samples are annotated (colored bars on the top) by type of samples (HV in pink, patients in cyan) and day after inclusion (blue scaled). Expression levels are color-coded from blue (low expression) to red (high expression). Similar patterns of expression are highlighted through hierarchical clustering of probesets (rows) with Euclidean distance and complete clustering method. **(A)** Expression levels in burns. **(B)** Expression levels in traumas. **(C)** Expression levels in septic shock 1. **(D)** Expression levels in septic shock 2. On each heatmap, the percentage of probesets with low intensity is shown.

Supplemental Figure 2 Most variant HERVs in severely injured patients. Venn diagram of the 84 most variant HERV probesets (25%) selected in each of the four datasets.

Supplemental Figure 3 Volcano plots of differentially expressed HERVs. **(A)** in burn cohort. **(B)** in trauma cohort. **(C)** in septic shock cohort 1 and **(D)** in septic shock cohort 2. The x-axis represents the log2 fold change between patient and HV, the y-axis the –log10 of adjusted *p*-values. Each point represents a probeset targeting HERV, in red the statistically differentially expressed between patients at D1 and HV. On each volcano plot, the number indicates the number of differentially expressed probesets.

Supplemental Figure 4 Heatmap representation of the modulated HERVs in severely injured patients at D1. Heatmap of the 56 differentially expressed probesets in at least 1 dataset. On the top bar, samples are color-coded in blue for HV and in red for Patients. On the bar below, samples are in green for Burn study, in yellow for Trauma study, in purple for Septic Shock 1 (SS1) study and in light red for Septic Shock 2 (SS2). Probesets are in rows and samples in columns. Expression levels from each cohort have been normalized (centered and reduced). Normalized expression levels are color-coded from blue (low expression) to red (high expression). Similar patterns of expression are highlighted through hierarchical clustering of probesets (rows) and samples (columns) with Euclidean distance and complete clustering method.

Supplemental Figure 5 Differentially expressed HERVs in severely injured patients. **(A)** Venn diagram of differentially expressed HERVs for each dataset. **(B)** Expression profiles of commonly modulated probesets targeting HERVs in the 4 datasets. Boxes are color-coded by day after inclusion. **(C)** Expression profiles of 2 selected probesets targeting HERVs. For each graphic, from top to bottom, title contains: probeset name, HERV name and closest gene.

Table S1 Clinical description of trauma cohort.

Table S2 Designs of qPCR systems. **(A)** Home-made designs. **(B)** Commercial designs.

Table S3 Differentially expressed HERVs. The 56 differentially expressed probesets targeting HERVs between patients at D1 and HV, in at least 1 dataset. Each tab correspond to a dataset. Each table shows the probeset names, the log2 fold changes, the Average Expression, the moderated t-statistic, the raw *p*-value, the adjusted *p*-value and the log-odds that the gene is differentially expressed.

## Abbreviations

HERV: Human endogenous retrovirus
LTR: Long Terminal Repeats
PAMP: pathogen-associated molecular pattern
LPS: Lipopolysaccharide
PMA: phorbol-12-myristate-13-acetate
HV: healthy volunteers
ICU: Intensive Care Unit
TBSA: Total Burn Surface Area
ABSI: Abbreviated Burn Severity Index
ISS: Injury Severity Score
SAPSII: Simplified Acute Physiology Score II
MFI: Medians of Fluorescence Intensity.

## References

[1] Young GR Stoye JP Kassiotis G. Are human endogenous retroviruses pathogenic? An approach to testing the hypothesis. Bioessays (2013) 35:794–803. 10.1002/bies.201300049 23864388 PMC4352332

[2] Cohen CJ Lock WM Mager DL. Endogenous retroviral LTRs as promoters for human genes: a critical assessment. Gene (2009) 448:105–14. 10.1016/j.gene.2009.06.020 19577618

[3] Isbel L Whitelaw E. Endogenous retroviruses in mammals: an emerging picture of how ervs modify expression of adjacent genes. BioEssays (2012) 34:734–8. 10.1002/bies.201200056 22736292

[4] Jern P Coffin JM. Effects of retroviruses on host genome function. Ann Rev Genet. (2008) 42:709–32. 10.1146/annurev.genet.42.110807.091501 18694346

[5] Mager DL Hunter DG Schertzer M Freeman JD. endogenous retroviruses provide the primary polyadenylation signal for two new human genes (HHLA2 and HHLA3). Genomics (1999) 59:255–63. 10.1006/geno.1999.5877 10444326

[6] Dunn CA Mager DL. Transcription of the human and rodent SPAM1 / PH-20 genes initiates within an ancient endogenous retrovirus. BMC Genomics (2005) 6:47. 10.1186/1471-2164-6-47 15804358 PMC1079825

[7] Gimenez J Montgiraud C Pichon JP Bonnaud B Arsac M Ruel K. Custom human endogenous retroviruses dedicated microarray identifies self-induced HERV-W family elements reactivated in testicular cancer upon methylation control. Nucleic Acids Res. (2010) 38:2229–46. 10.1093/nar/gkp1214 20053729 PMC2853125

[8] Pérot P Mullins CS Naville M Bressan C Hühns M Gock M. Expression of young HERV-H loci in the course of colorectal carcinoma and correlation with molecular subtypes. Oncotarget (2015) 6:40095–111. 10.18632/oncotarget.5539 26517682 PMC4741882

[9] Laska MJ Brudek T Nissen KK Christensen T Møller-Larsen A Petersen T. Expression of HERV-Fc1, a human endogenous retrovirus, is increased in patients with active multiple sclerosis. J Virol. (2012) 86:3713–22. 10.1128/JVI.06723-11 22278236 PMC3302483

[10] Balada E Vilardell-Tarrés M Ordi-Ros J. Implication of human endogenous retroviruses in the development of autoimmune diseases. Int Rev Immunol. (2010) 29:351–70. 10.3109/08830185.2010.485333 20635879

[11] Madeira A Burgelin I Perron H Curtin F Lang AB Faucard R. MSRV envelope protein is a potent, endogenous and pathogenic agonist of human toll-like receptor 4: relevance of GNbAC1 in multiple sclerosis treatment. J Neuroimmunol. (2016) 291:29–38. 10.1016/j.jneuroim.2015.12.006 26857492

[12] Young GR Eksmond U Salcedo R Alexopoulou L Stoye JP Kassiotis G. Resurrection of endogenous retroviruses in antibody-deficient mice. Nature (2012) 491:774–8. 10.1038/nature11599 23103862 PMC3511586

[13] Johnston JB Silva C Holden J Warren KG Clark AW Power C. Monocyte activation and differentiation augment human endogenous retrovirus expression: implications for inflammatory brain diseases. Ann Neurol. (2001) 50:434–42. 10.1002/ana.1131 11601494

[14] Lee YJ Jeong BH Park JB Kwon HJ Kim YS Kwak IS. The prevalence of human endogenous retroviruses in the plasma of major burn patients. Burns (2013) 39:1200–5. 10.1016/j.burns.2012.12.013 23339865

[15] Lee KH Rah H Green T Lee YK Lim D Nemzek J. divergent and dynamic activity of endogenous retroviruses in burn patients and their inflammatory potential. Exp Mol Pathol. (2014) 96:178–87. 10.1016/j.yexmp.2014.02.001 24509167 PMC4104748

[16] Plassais J Venet F Cazalis MA Le Quang D Pachot A Monneret G. Transcriptome modulation by hydrocortisone in severe burn shock: ancillary analysis of a prospective randomized trial. Critical Care (2017) 21:158. 10.1186/s13054-017-1743-9 28623938 PMC5473974

[17] Xiao W Mindrinos MN Seok J Cuschieri J Cuenca AG Gao H. A genomic storm in critically injured humans. J Exp Med. (2011) 208:2581–90. 10.1084/jem.20111354 22110166 PMC3244029

[18] Ong DSY Bonten MJM Spitoni C Verduyn Lunel FM Frencken JF Horn J. Epidemiology of multiple herpes viremia in previously immunocompetent patients with septic shock. Clin Infect Dis. (2017) 64:1204–10. 10.1093/cid/cix120 28158551

[19] Textoris J Mallet F Immunosuppression and herpes viral reactivation in intensive care unit patients: one size does not fit all. Crit Care (2017) 21:230 10.1186/s13054-017-1803-1 28841888 PMC5574101

[20] Walton AH Muenzer JT Rasche D Boomer JS Sato B Brownstein BH. Reactivation of multiple viruses in patients with sepsis. PLoS ONE (2014) 9:e98819. 10.1371/journal.pone.0098819 24919177 PMC4053360

[21] Young GR Mavrommatis B Kassiotis G. Microarray analysis reveals global modulation of endogenous retroelement transcription by microbes. Retrovirology (2014) 11:59. 10.1186/1742-4690-11-59 25063042 PMC4222864

[22] Reichmann J Crichton JH Madej MJ Taggart M Gautier P Garcia-Perez JL. Microarray analysis of LTR retrotransposon silencing identifies Hdac1 as a regulator of retrotransposon expression in mouse embryonic stem cells. PLOS Comput Biol. (2012) 8:e1002486. 10.1371/journal.pcbi.1002486 22570599 PMC3343110

[23] Venet F Plassais J Textoris J Cazalis MA Pachot A Bertin-Maghit M. Low-dose hydrocortisone reduces norepinephrine duration in severe burn patients: a randomized clinical trial. Crit Care (2015) 19:21. 10.1186/s13054-015-0740-0 25619170 PMC4347659

[24] Gouel-Chéron A Allaouchiche B Floccard B Rimmelé T Monneret G. Early daily MHLA-DR monitoring predicts forthcoming sepsis in severe trauma patients. Intens Care Med. (2015) 41:2229–30. 10.1007/s00134-015-4045-1 26359166

[25] Cazalis MA Lepape A Venet F Frager F Mougin B Vallin H. Early and dynamic changes in gene expression in septic shock patients: a genome-wide approach. Intens Care Med Exp. (2014) 2:20. 10.1186/s40635-014-0020-3 26215705 PMC4512996

[26] Venet F Schilling J Cazalis MA Demaret J Poujol F Girardot T. Modulation of LILRB2 protein and MRNA expressions in septic shock patients and after ex vivo lipopolysaccharide stimulation. Hum Immunol. (2017) 78:441–50. 10.1016/j.humimm.2017.03.010 28341250

[27] Wilson CL Miller CJ. Simpleaffy: a bioconductor package for affymetrix quality control and data analysis. Bioinformatics (2005) 21:3683–85. 10.1093/bioinformatics/bti605 16076888

[28] Wu Z Irizarry RA. Stochastic models inspired by hybridization theory for short oligonucleotide arrays. J Comput Biol. (2005) 12:882–93. 10.1089/cmb.2005.12.882 16108723

[29] Johnson WE Li C Rabinovic A. Adjusting batch effects in microarray expression data using empirical bayes methods. Biostatistics (2007) 8:118–27. 10.1093/biostatistics/kxj037 16632515

[30] Ritchie ME Phipson B Wu D Hu Y Law CW Shi W. Limma powers differential expression analyses for RNA-sequencing and microarray studies. Nucleic Acids Res. (2015) 43:e47. 10.1093/nar/gkv007 25605792 PMC4402510

[31] Benjamini Y Hochberg Y Controlling the false discovery rate: a practical and powerful approach to multiple testing. J R Stat Soc Series B (1995) 57:289–300.

[32] Friggeri A Cazalis MA Pachot A Cour M Argaud L Allaouchiche B. Decreased CX3CR1 messenger rna expression is an independent molecular biomarker of early and late mortality in critically Ill patients. Critical Care (2016) 20:204. 10.1186/s13054-016-1362-x 27364780 PMC4929760

[33] Aken BL Achuthan P Akanni W,M Amode R Bernsdorff F Ensembl 2017. Nucleic Acids Res. (2017) 45:D635–42. 10.1093/nar/gkw1104 27899575 PMC5210575

[34] Gimenez JLG Carbonell EN Mateo CR López EG Palacios L Chova LP Epigenetics as the driving force in long-term immunosuppression. J Clin Epigenet. (2016) 2:2 10.21767/2472-1158.100017

[35] Saeed S Quintin J Kerstens HH Rao NA Aghajanirefah A Matarese F. Epigenetic programming of monocyte-to-macrophage differentiation and trained innate immunity. Science (2014) 345:1251086. 10.1126/science.1251086 25258085 PMC4242194

[36] Daskalakis M Brocks D Sheng Y -H Islam S Ressnerova A Assenov Y Reactivation of endogenous retroviral elements via treatment with DNMT- and HDAC-inhibitors. Cell Cycle (2018) 0:1–12. 10.1080/15384101.2018.1442623 PMC6056222 29633898

[37] Lamprecht B Walter K Kreher S Kumar R Hummel M Lenze D. Derepression of an endogenous long terminal repeat activates the CSF1R proto-oncogene in human lymphoma. Nat Med. (2010) 16:571–9. 10.1038/nm.2129 20436485

[38] Beyer U Krönung SK Leha A Walter L Dobbelstein M. Comprehensive identification of genes driven by ERV9-LTRs reveals TNFRSF10B as a re-activatable mediator of testicular cancer cell death. Cell Death Differ. (2016) 23:64–75. 10.1038/cdd.2015.68 26024393 PMC4815979

[39] Chiappinelli KB Strissel PL Desrichard A Li H Henke C Akman B. Inhibiting DNA methylation causes an interferon response in cancer via dsrna including endogenous retroviruses. Cell (2015) 162:974–86. 10.1016/j.cell.2015.07.011 26317466 PMC4556003

[40] Groh S Schotta G. Silencing of endogenous retroviruses by heterochromatin. Cell Mol Life Sci. (2017) 74:2055–65. 10.1007/s00018-017-2454-8 28160052 PMC11107624

[41] Chuong EB Elde NC Feschotte C. Regulatory evolution of innate immunity through co-option of endogenous retroviruses. Science (2016) 351:1083–7. 10.1126/science.aad5497 26941318 PMC4887275

[42] Alvarez-Errico D Aguilar H Kitzig F Brckalo T Sayós J López-Botet M. IREM-1 Is a novel inhibitory receptor expressed by myeloid cells. Eur J Immunol. (2004) 34:3690–701. 10.1002/eji.200425433 15549731

[43] Becker J Pérot P Cheynet V Oriol G Mugnier N Mommert M. A comprehensive hybridization model allows whole HERV transcriptome profiling using high density microarray. BMC Genomics (2017) 18:286. 10.1186/s12864-017-3669-7 28390408 PMC5385096

