# Supplementary figures and images for: Endogenous Retroviruses Transcriptional Modulation After Severe Infection, Trauma and Burn
Source: Front Immunol. 2019 Jan 8;9:3091. doi: 10.3389/fimmu.2018.03091 (PMC6331457; doi:10.3389/fimmu.2018.03091)

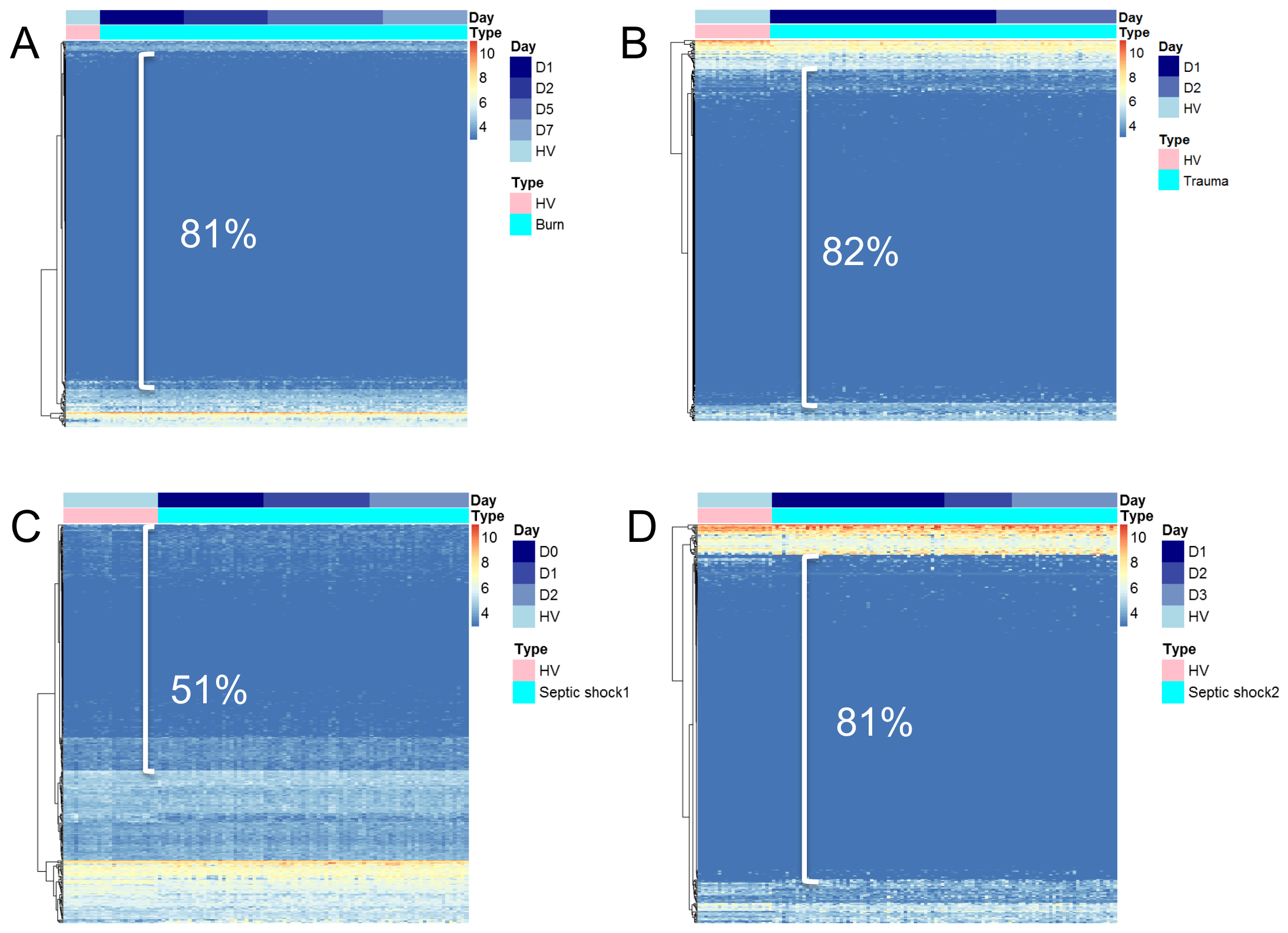

Supplement: Supplemental Figure 1 — Heatmap representation of HERVs in three models of injury. Heatmap of the 337 probesets targeting HERVs in the four datasets: burn, trauma and 2 septic shock cohorts. Probesets are in rows and samples in columns. Samples are annotated (colored bars on the top) by type of samples (HV in pink, patients in cyan) and day after inclusion (blue scaled). Expression levels are color-coded from blue (low expression) to red (high expression). Similar patterns of expression are highlighted through hierarchical clustering of probesets (rows) with Euclidean distance and complete clustering method. (A) Expression levels in burns. (B) Expression levels in traumas. (C) Expression levels in septic shock 1. (D) Expression levels in septic shock 2. On each heatmap, the percentage of probesets with low intensity is shown. [file Image_1.TIF]

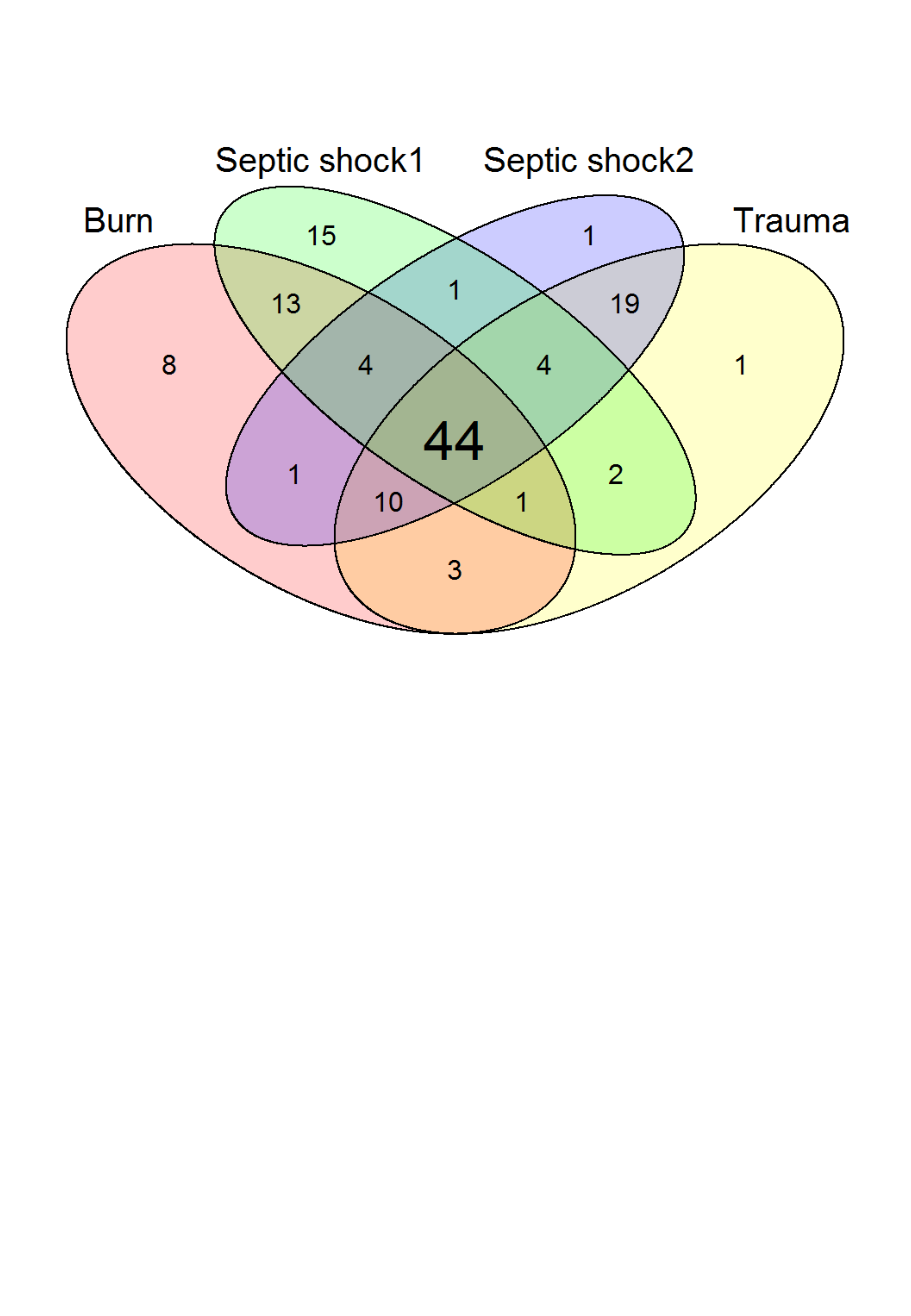

Supplement: Supplemental Figure 2 — Most variant HERVs in severely injured patients. Venn diagram of the 84 most variant HERV probesets (25%) selected in each of the four datasets. [file Image_2.TIF]

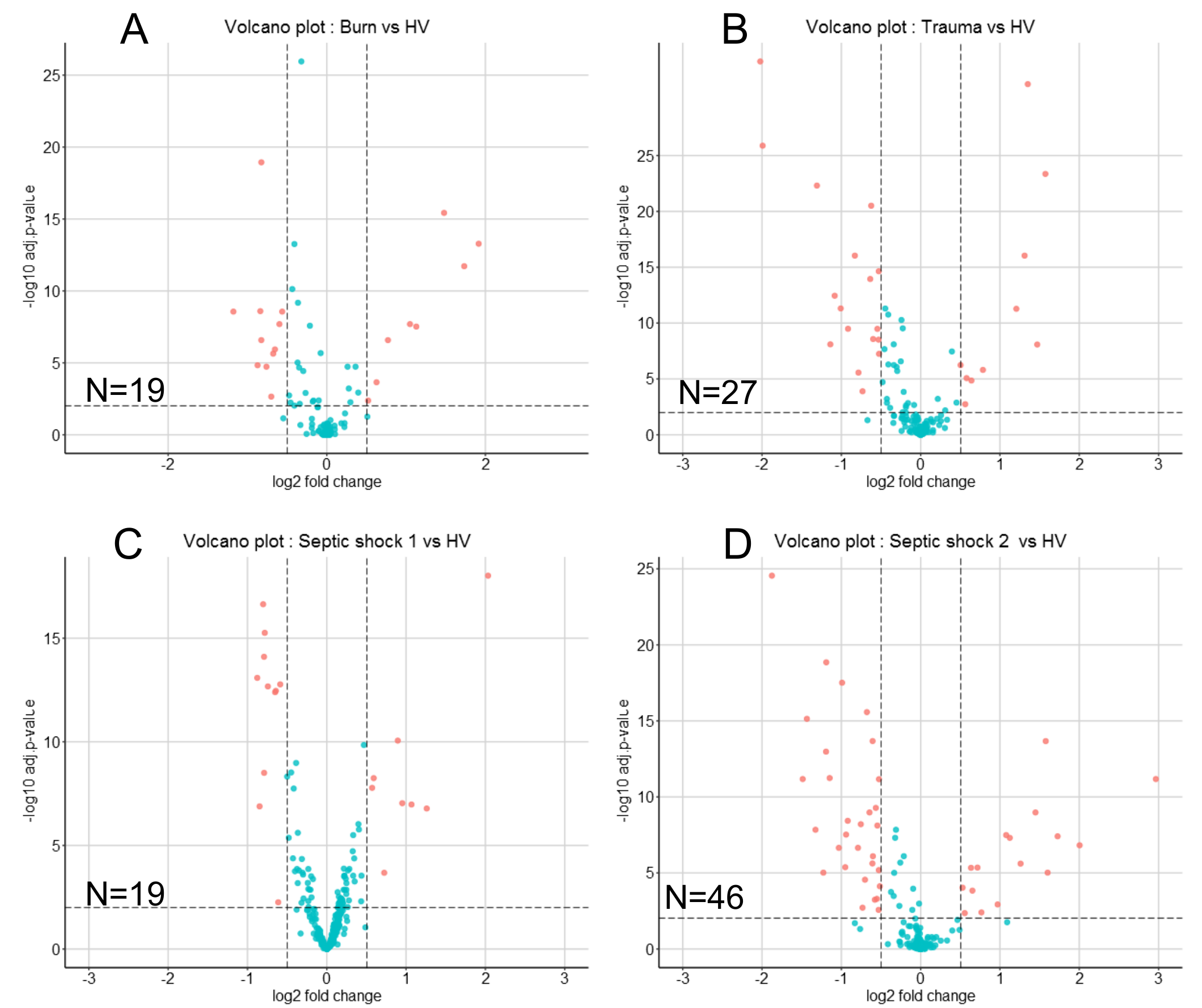

Supplement: Supplemental Figure 3 — Volcano plots of differentially expressed HERVs. (A) in burn cohort. (B) in trauma cohort. (C) in septic shock cohort 1 and (D) in septic shock cohort 2. The x-axis represents the log2 fold change between patient and HV, the y-axis the –log10 of adjusted p-values. Each point represents a probeset targeting HERV, in red the statistically differentially expressed between patients at D1 and HV. On each volcano plot, the number indicates the number of differentially expressed probesets. [file Image_3.TIF]

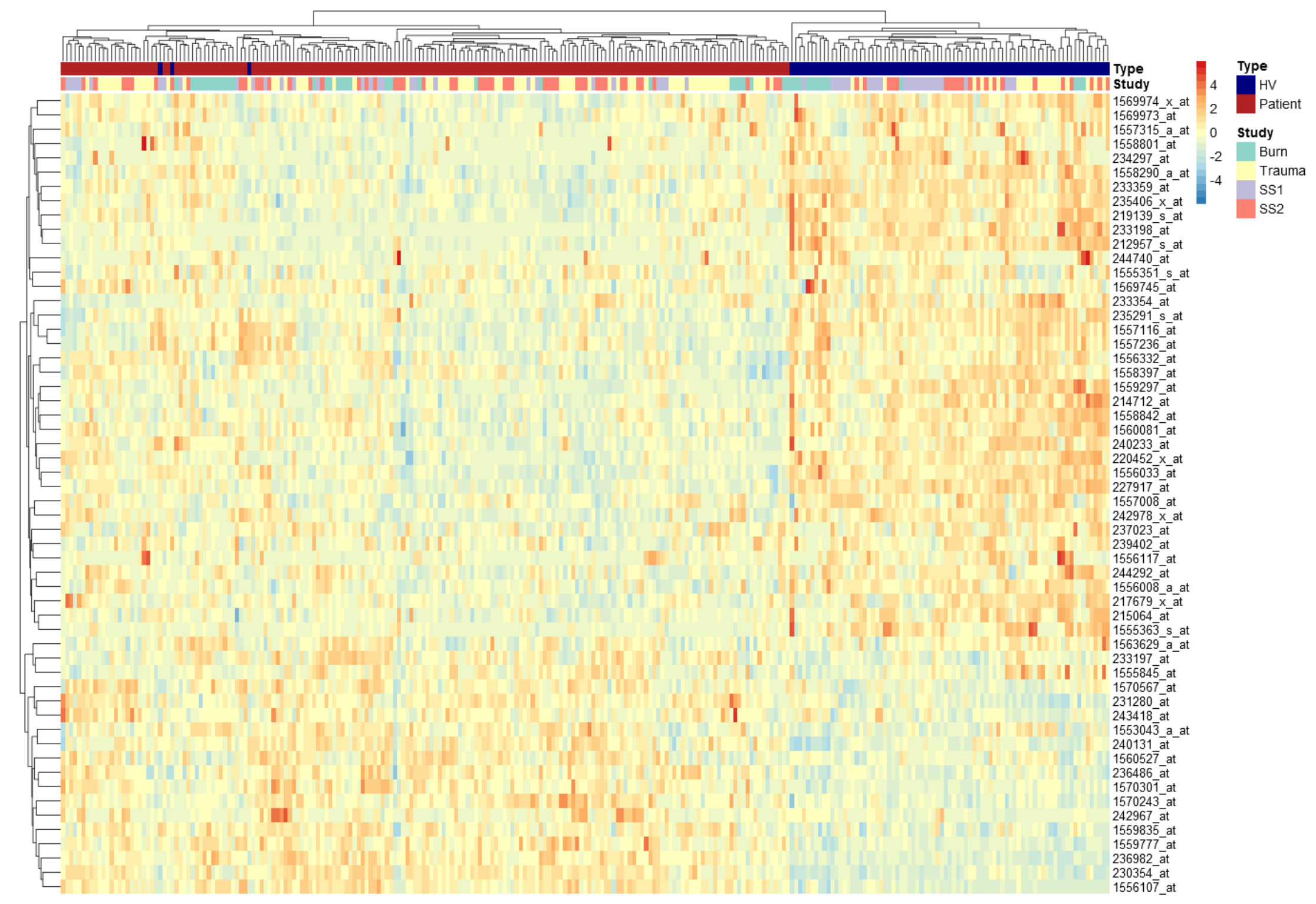

Supplement: Supplemental Figure 4 — Heatmap representation of the modulated HERVs in severely injured patients at D1. Heatmap of the 56 differentially expressed probesets in at least 1 dataset. On the top bar, samples are color-coded in blue for HV and in red for Patients. On the bar below, samples are in green for Burn study, in yellow for Trauma study, in purple for Septic Shock 1 (SS1) study and in light red for Septic Shock 2 (SS2). Probesets are in rows and samples in columns. Expression levels from each cohort have been normalized (centered and reduced). Normalized expression levels are color-coded from blue (low expression) to red (high expression). Similar patterns of expression are highlighted through hierarchical clustering of probesets (rows) and samples (columns) with Euclidean distance and complete clustering method. [file Image_4.TIF]

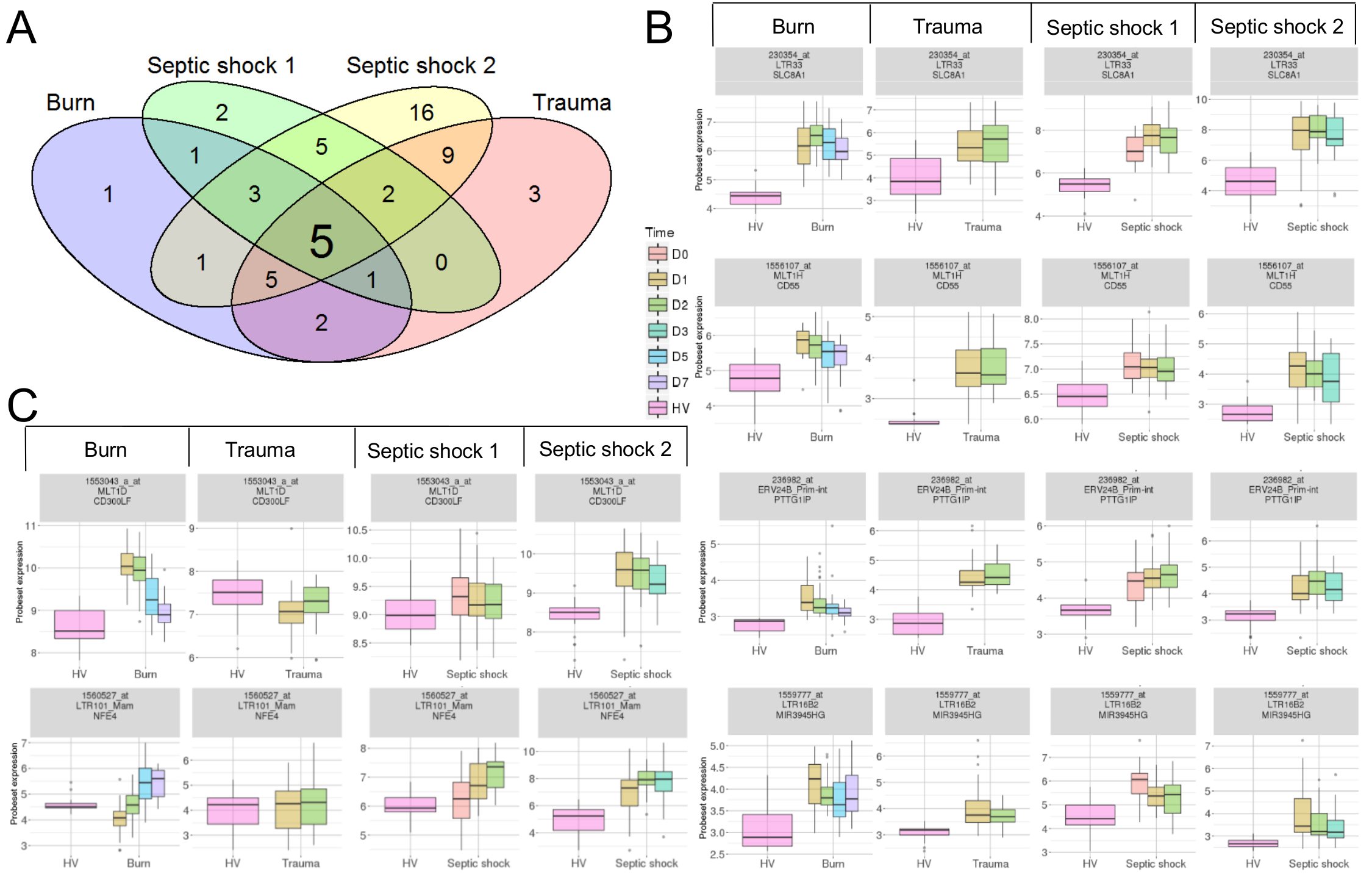

Supplement: Supplemental Figure 5 — Differentially expressed HERVs in severely injured patients. (A) Venn diagram of differentially expressed HERVs for each dataset. (B) Expression profiles of commonly modulated probesets targeting HERVs in the 4 datasets. Boxes are color-coded by day after inclusion. (C) Expression profiles of 2 selected probesets targeting HERVs. For each graphic, from top to bottom, title contains: probeset name, HERV name and closest gene. [file Image_5.TIF]
